# In Vitro Antiviral Activity of *Kalanchoe daigremontiana* Extract against Human Herpesvirus Type 1

**DOI:** 10.3390/ijms25147507

**Published:** 2024-07-09

**Authors:** Marcin Chodkowski, Sylwia Nowak, Martyna Janicka, Marcin Sobczak, Sebastian Granica, Marcin W. Bańbura, Malgorzata Krzyzowska, Joanna Cymerys

**Affiliations:** 1 Division of Medical and Environmental Microbiology, Military Institute of Hygiene and Epidemiology, Kozielska 4, 01-163 Warsaw, Poland; martyna.janicka@wihe.pl (M.J.); malgorzata.krzyzowska@wihe.pl (M.K.); 2 Division of Microbiology, Department of Preclinical Sciences, Institute of Veterinary Medicine, Warsaw University of Life Sciences, 02-786 Warsaw, Poland; marcin_banbura@sggw.edu.pl; 3 Helpcosmetics Sp.z o.o. Al. Zwycięstwa 241/13, 81-521 Gdynia, Poland; s.nowak@helpcosmetics.pl; 4 Department of Pharmaceutical Chemistry and Biomaterials, Faculty of Pharmacy, Medical University of Warsaw, 1 Banacha Street, 02-097 Warsaw, Poland; marcin.sobczak@wum.edu.pl; 5 Department of Pharmaceutical Biology, Medical University of Warsaw, 02-097 Warsaw, Poland; sebastian.granica@wum.edu.pl

**Keywords:** *Kalanchoe daigremontiana*, human herpesvirus type 1, HHV-1, plant medicine

## Abstract

Plant polyphenols possess diverse bioactivities, including antiviral activity against a broad spectrum of viruses. Here, we investigated the virucidal properties of an *Kalanchoe daigremontiana* extract using an in vitro model of human herpesvirus type 1 (HHV-1) infection. Chromatographic analysis indicated that the extract of *Kalanchoe daigremontiana* is rich in various compounds, among which are polyphenols with virucidal activity confirmed in the literature. We found that *Kalanchoe daigremontiana* extract shows an ability to prevent HHV-1 infection by direct inhibition of the virus attachment, penetration, and blocking of infection when used in pretreatment or post-entry treatment. Our results indicate that *Kalanchoe daigremontiana* extract may be a good candidate drug against HHV-1, both as a substance to prevent infection and to treat an already ongoing infection. Our findings illustrate that *Kalanchoe daigremontiana* could be a potential new candidate for clinical consideration in the treatment of HHV-1 infection alone or in combination with other therapeutics.

## 1. Introduction

**Figure 1:**
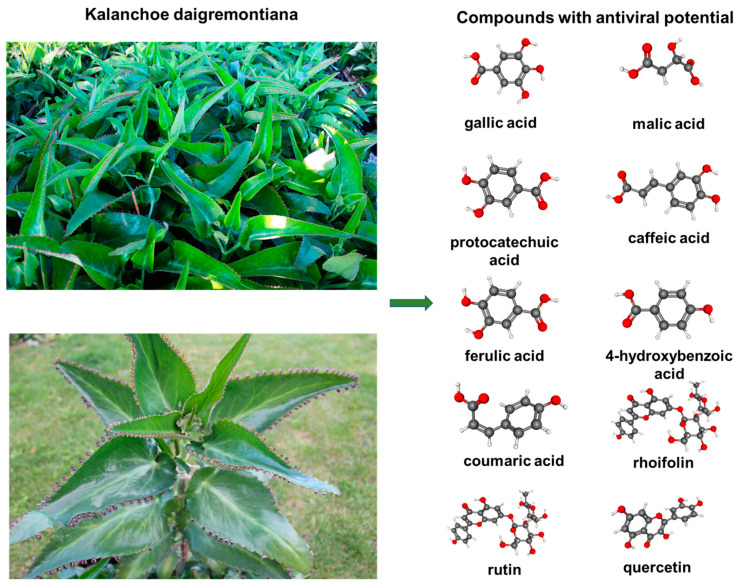
Images of *Kalanchoe daigremontiana* with a list of some compounds with virucidal potential based on chromatographic analysis. Photos by Dr. Sylwia Nowak; models of compounds - gray indicates carbon atoms, red indicates oxygen and white indicates hydrogen atoms; https://pubchem.ncbi.nlm.nih.gov/, accessed on 10 May 2024. Components with potential antiviral activity were selected on the basis of literature data [5,6,7,8,9,10,11,12,13,14].

Plants are the source of many substances with therapeutic potential. Increasingly, we are returning to natural medicine in search of alternatives for the treatment of various diseases [1]. Plant medicine is very popular for the treatment of minor ailments in societies with difficult access to medical help. *Kalanchoe daigremontiana* Raym.-Hamet and H. Perrier, also named “mother of thousands”, is a succulent originating from Madagascar [2]. This plant is widely distributed in North and South America, growing up to about one meter. Because of its rapid and easy spread, it is classified as an invasive species. The cultivation and research of kalanchoe is becoming increasingly popular due to its broad bioactivities. Antitumor, antimicrobial, and anti-inflammatory properties of *K. daigremontiana* extracts in the treatment of gastric diseases, but also in disorders of the nervous system, such as anxiety and restlessness, have been described in the literature. The biological properties of *Kalanchoe* species extracts are determined by the presence of numerous compounds, such as polyphenols and bufadienolides [3]. The plant is also rich in phenolic acids such as gallic, chlorogenic, ferulic, caffeic, and p-coumaric acids (Figure 1). The content of compounds in the extracts depends on the cultivation conditions and the extraction method [3]. The antiviral properties of *K. daigremontiana* extracts have not been extensively studied. So far, only *Kalanchoe pinniata* extracts and their components have been proven to show antiviral properties [2,4].

Plant polyphenols are naturally occurring phytochemicals with different structures and properties that protect plants from infections, environmental stresses, etc. Their ability to inhibit viral infections is exhibited by their direct interaction with viral particles or by their inhibition of excessive oxidative stress caused by some viruses [5,6]. The antiviral activity of both flavonoid and non-flavonoid polyphenols against HHV-1 and HHV-2 (human herpes virus type 1 and 2; herpes simplex virus type 1 and 2) has been described. The flavonoids exerting anti-HHV activity include quercetin, kaempferol, luteolin, myrecitin, epicatechin, epigallocatechin, and genistein [6,7,8,9,10]. Non-flavonoid polyphenols with proven antiviral activity in HHV infections belong to phenolic acids (ginkgolic acid, ellagic acid, trans-ferulic acid, gentisic acid, vanillic acid, syringic acid, and gallic acid), tannins and their derivatives (tannic acid, punicalagin, pentagalloylglucose), stilbenes (resveratrol), xanthones (mangiferin), curcuminoids (curcumin), coumarins, and furanocoumarins [7,8,9,10].

The World Health Organization (WHO) estimates that over 4 billion people are infected with HHV-1 (3.7 billion cases) and HHV-2 (400 million cases) [11]. Infection caused by HHV is lifelong, due to its ability to establish a state of latency in nerve cell bodies. Reactivation of the infection usually occurs several times a year in situations of immunosuppression or stress. Herpesvirus infection is commonly associated with cold sores, genital ulceration, encephalitis, and corneal blindness [11]. Mortality rates of HHV encephalitis are between 5 and 20%, depending on the immune status, treatment used, age, and co-morbidities [11]. Moreover, the inflammatory changes in the CNS caused by herpes simplex encephalitis may lead to development of autoimmune encephalitis (AE) within 3 months of HHV encephalitis in up to 27% of cases [12]. Available antiviral therapies, such as acyclovir, penciclovir, famciclovir, cidofovir, valacyclovir, trifluridine, and vidarabine are based on the inhibition of viral polymerase. Given the increasing resistance of HHV strains to nucleoside analogs, there is a need to search for substances based on other mechanisms of action [13]. However, there are no effective drugs that inhibit the spread of the virus to neighboring cells. In addition, there is still no drug proposed to destroy the virus in its latent state. There are also no effective vaccines available, despite several expression models proposed. Due to the increasing resistance of the strains to available therapies and the lack of effective vaccines, there is a need to search for new substances with antiviral potential, which would also stimulate the immune system to fight the infection. The following work demonstrates the virucidal effect under in vitro conditions using a plant extract derived from *K. daigremontiana*. We believe that the active substances that are components of the extracts or the extracts from *K. daigremontiana* themselves can be used in the treatment of the human herpesvirus type 1 infection.

## 2. Results

### 2.1. Chemical Characteristics of the Extract from K. daigremontiana

**Figure 2:**
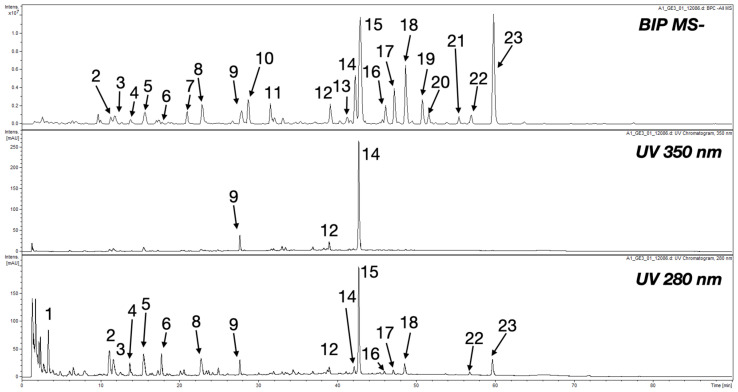
UHPLC-DAD-MS chromatograms of *K. daigremontiana* extract.

UHPLC-DAD-MS analysis identified 23 compounds (Figure 2, Table 1). The results of the analysis of the water extract from *K. daigremontiana* are shown in Table 1. Most of them were kaempferol glucosides (**8**, and **15**), others included glycoside of quercetin (**12**), bersaldegenin acetates (**16**, **17**, and **23**), daigremontianin derivatives (**13**, and **18**), and bryophyllin (**14**). Three flavonoids and eleven compounds were not identified based on obtained chromatographic data (N.I.) (Figure 2, Table 1).

### 2.2. Effects of Aqueous Extract of K. daigremontiana on Cell Viability

**Figure 3:**
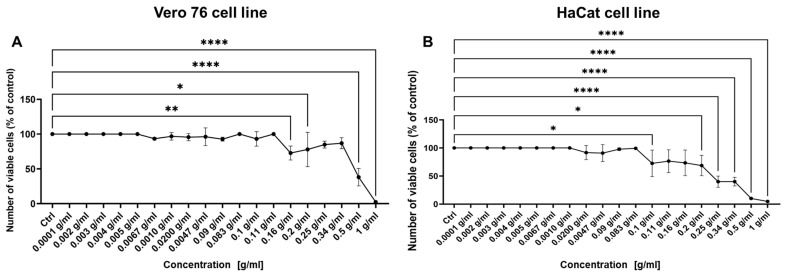
Cell viability after exposure to *K. daigremontiana* extract was evaluated using cell proliferation WST-1 assay: (**A**) Vero 76 cell line; (**B**) HaCaT cell line. Data from three independent experiments are presented as mean ± SEM. Tukey’s multiple comparison test with * *p* ≤ 0.05, ** *p* ≤ 0.01, and extremely significant **** *p* ≤ 0.0001.

The cytotoxicity of *K. daigremontiana* extract on epithelial cells (Vero and HaCaT cells) was determined via a WST-1 assay (tetrazolium salt-based test). The results of the WST-1 assay showed that the extract was more toxic at higher concentrations to HaCaT cells than to Vero cells. The IC 50 values were for Vero 76 cell line—0.5 g/mL, and for HaCaT cells—0.25 g/mL (Figure 3). Accordingly, non-cytotoxic concentrations of *K. daigremontiana* extract were used to determine its antiviral activity.

For in vitro antiviral assays we decided to use *K. daigremontiana* extract with the concentration range of 0.0001 to 0.16 g/mL (maximum non-toxic concentrations, MNTC). 

### 2.3. Antiviral Assays with Extract of K. daigremontiana against HHV-1

**Figure 4:**
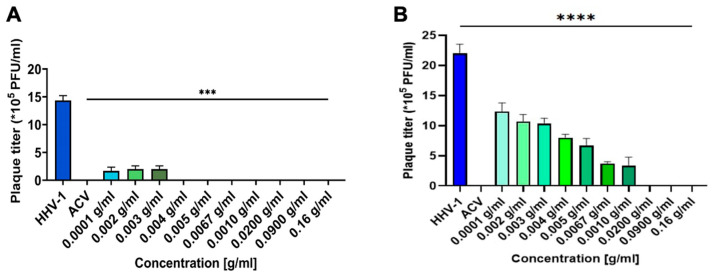
The anti-HHV-1 activity of *K. diagremontiana* extract. Virus titration in Vero 76 cells (**A**) and HaCat cells (**B**) (24 h p.i.). Data from three independent experiments are presented as mean ± SEM. Tukey’s multiple comparison test with extremely significant at *** *p* ≤ 0.001 or **** *p* ≤ 0.0001.

A viral plaque assay showed that *K. daigremontiana* extract significantly inhibited the plaque formation of HSV-1 in Vero cells within the tested concentration range (Figure 4A). To evaluate PFU in the HaCat cell line, we collected cell media from over HaCat cells infected and treated with different concentrations of the extract and added them to the Vero 76 cell line. Similarly, we observed a significant decrease in PFU (Figure 4B).

**Figure 5:**
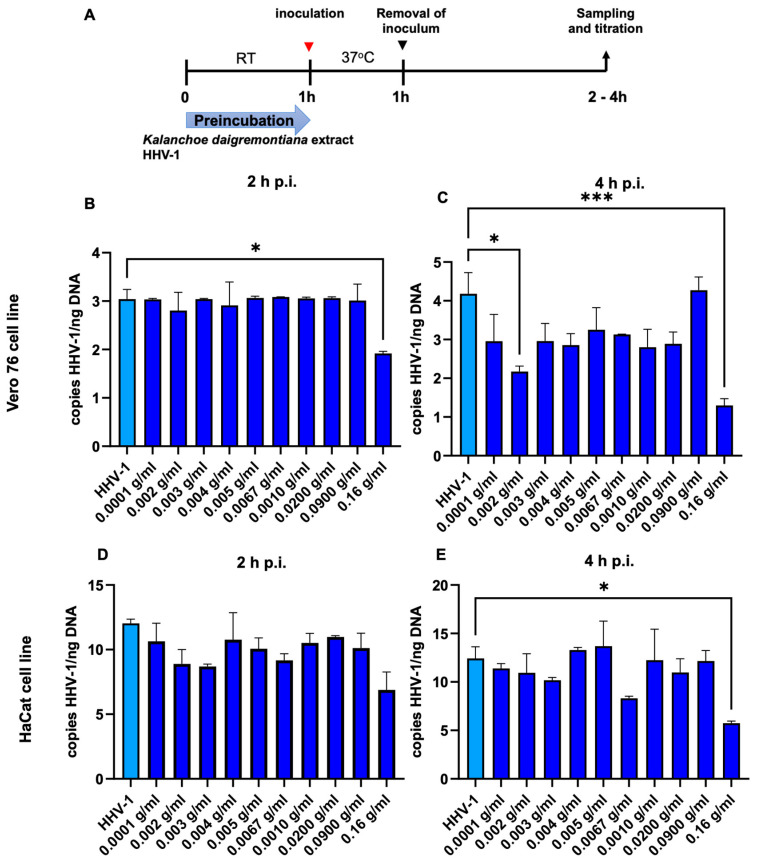
*K. daigremontiana* extract blocks HHV-1 entry into the Vero 76 and HaCaT cells. Schematic representation of test procedures (**A**); inhibition of virus entry in Vero76 (**B**,**C**) and HaCaT (**D**,**E**) cell cultures with *K. daigremontiana* extract. At 2 and 4 h p.i., cells were subjected to HHV-1 copies titration by qPCR. Data from three independent experiments are presented as means ± SEM. Tukey’s multiple comparison test * *p* ≤ 0.05, and extremely significant at *** *p* ≤ 0.001.

To investigate whether *K. daigremontiana* extract blocks virus entry (Figure 5), we checked the amount of viral DNA at 2 and 4 h after infection. In the Vero 76 cell line, two hours after infection (Figure 5B), only a concentration of 0.16 g/mL statistically reduced viral entry (*p* ≤ 0.05). Four hours after infection (Figure 5C), we already observed a decrease in all concentrations of the extract, where statistically significant results were obtained for concentrations of 0.002 g/mL and 50% less viral DNA was observed (*p* ≤ 0.05). On the other hand, at a concentration of 0.16 g/mL, we observed a threefold decrease in the amount of viral DNA, compared to the infected, untreated *K. daigremontiana* extract (*p* ≤ 0.001). 

In the HaCat cell line, we also observed less viral DNA in the cells 2 h after infection, but these were not statistically significant. We observed the greatest decrease at an extract concentration of 0.16 g/mL. This trend also continued 4 h after infection (*p* ≤ 0.050) (Figure 5D,E). 

**Figure 6:**
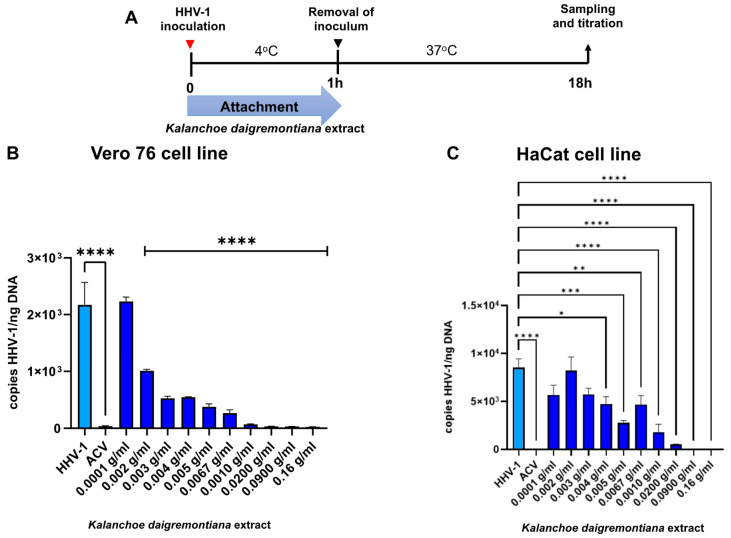
*K. diagremontiana* extract inhibits HHV-1 attachment in Vero 76 and HaCaT cells. Schematic representation of test procedures (**A**); inhibition of virus attachment in Vero76 (**B**) and HaCaT (**C**) cell cultures with *K. daigremontiana* extract. At 24 h p.i., cells were subjected to HHV-1 copies titration by qPCR. Data from three independent experiments are presented as means ± SEM. Tukey’s multiple comparison test * *p* ≤ 0.05, ** *p* ≤ 0.01, and extremely significant at *** *p* ≤ 0.001 or **** *p* ≤ 0.0001.

**Figure 7:**
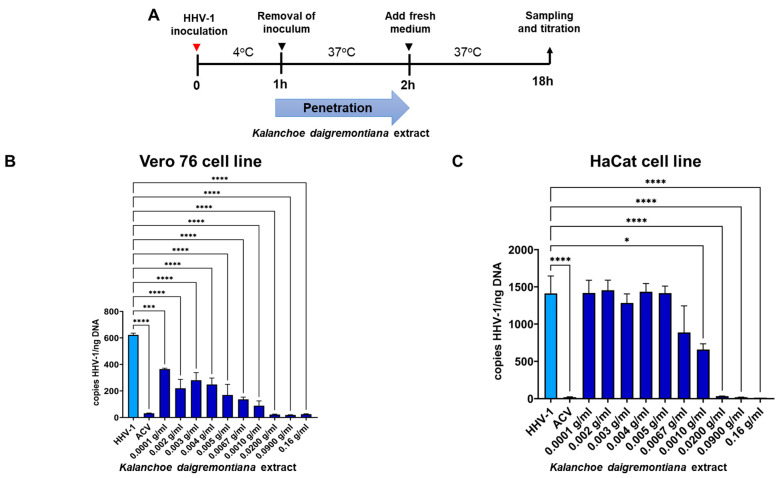
*K. diagremontiana* extract inhibits HHV-1 penetration in Vero 76 and HaCaT cells. Schematic representation of test procedures (**A**); inhibition of virus penetration in Vero (**B**) and HaCaT (**C**) cell cultures with *K. daigremontiana* extract. At 24 h p.i., cells were subjected to HHV-1 copies titration by qPCR. Data from three independent experiments are presented as mean ± SEM. Tukey’s multiple comparison test * *p* ≤ 0.05, and extremely significant at *** *p* ≤ 0.001 or **** *p* ≤ 0.0001.

To determine the antiviral effects of the *K. daigremontiana* extract, we performed viral attachment and penetration assays. A diagram showing the attachment and penetration test is given in Figure 6A and Figure 7A. *K. daigremontiana* extract inhibits virus attachment stronger in the Vero 76 cell line compared to the HaCaT cells (Figure 6B). At the concentration range of 0.005 to 0.16 g/mL, there was an almost 90% reduction in the virus copies (*p* ≤ 0.0001). Similar results were obtained for cells treated with ACV (Figure 6B,C). When viral copies were measured in culture medium, no significant differences were observed between infected cells and cells treated with the extract. For HaCaT cells, (Figure 6C), attachment inhibition was generally weaker—it was most strongly inhibited by extract concentrations ranging from 0.001 to 0.16 g/mL (*p* ≤ 0.0001) (Figure 6).

Then, we evaluated how the extract affects the fusion of the virus with the cell membrane (penetration assay). A greater penetration inhibition was obtained in the Vero cell line compared to the HaCaT cells (Figure 7B) (*p* ≤ 0.0001). As in the attachment assay, a dose-dependency in the inhibition of HHV-1 replication was observed. For the HaCaT cell line (Figure 7C), a statistically significant decrease was observed for the extract concentrations in the penetration assay starting from 0.0067 g/mL (*p* ≤ 0.05) (Figure 7). 

**Figure 8:**
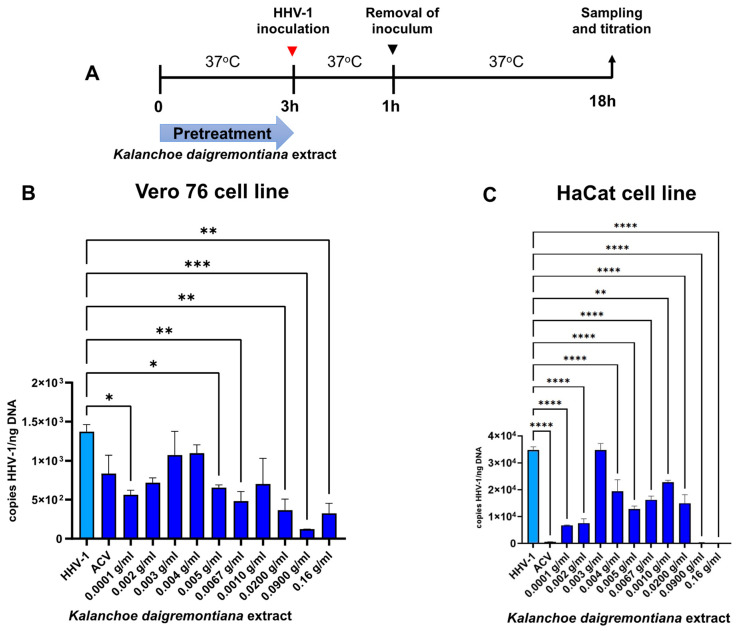
Efficiency of pretreatment with *K. daigremontiana* extract. Schematic representation of test procedures (**A**); Vero76 cell line (**B**) and HaCaT cell line (**C**). At 24 h p.i., cells were subjected to HHV-1 copies titration by qPCR. Data from three independent experiments are presented as mean ± SEM. Tukey’s multiple comparison test * *p* ≤ 0.05, ** *p* ≤ 0.01, and extremely significant at *** *p* ≤ 0.001 or **** *p* ≤ 0.0001.

A pretreatment test was performed to investigate the potential blocking of cell receptors by the extract components. For this purpose, we incubated the cell cultures with the extract 3 h before infection (Figure 8A). 

Prior application of the *K. daigremontiana* extract has a greater inhibitory effect on HHV-1 replication in the HaCaT cell line (Figure 8C). Furthermore, the efficiency of infection inhibition is observed at the lowest extract concentrations of 0.0001–0.002 g/mL (*p* ≤ 0.0001). The higher doses of extract inhibited HHV-1 replication by more than 90% (*p* ≤ 0.0001) (Figure 8C). In the Vero cell line, we also observed a decrease in HHV-1 replication, but it was less pronounced compared to that of the HaCaT cell line. The most effective concentration of extract in the pretreatment assay for the Vero cell line was 0.009 g/mL (*p* ≤ 0.001) (Figure 8B).

**Figure 9:**
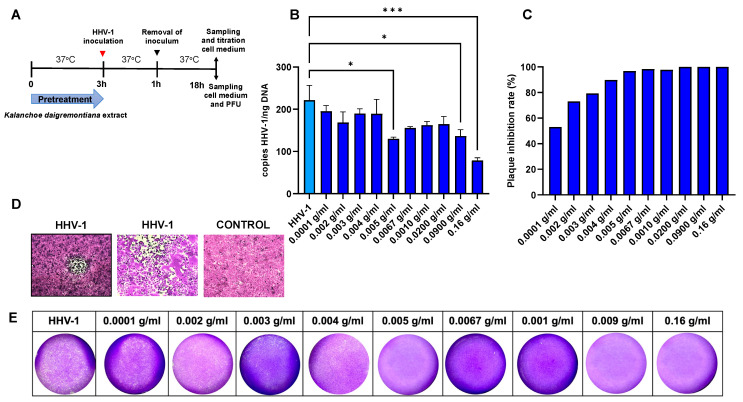
Efficiency of pretreatment with *K. daigremontiana* extract for the release of effective virions. Schematic representation of test procedures (**A**); At 24 h p.i., cells media from Vero 76 were subjected to HHV-1 copies titration by qPCR (**B**). Plaque inhibition rate on Vero 76 cell line. The experiment used cell media from culture-treated *K. daigremontiana* (**C**). Representative images of the cytopathic effect caused by HHV-1—plaque and diffuse cytopathic effect (microscope magnification 400×) (**D**). Representative images of crystal violet-stained HSV-1-infected Vero 76 cultures with visible cytopathic effect (PFU) wells, treated *K. daigremontiana* extracts (microscope magnification 100×) (**E**). Data from three independent experiments are presented as mean ± SEM. Tukey’s multiple comparison test * *p* ≤ 0.05, and extremely significant at *** *p* ≤ 0.001.

In addition, we tested whether administration of the extract before infection affects the release of effective virions capable of infection. For this purpose, we measured the number of viral copies in the culture medium and performed a standard PFU assay (Figure 9). We observed a statistically significant decrease in the number of viral copies in the culture medium compared to the sample not treated with the extract. We observed the most effective decrease at the concentration of 0.16 g/mL (*p* ≤ 0.001), and the statistically significant decrease was observed at concentrations of 0.005 and 0.09 g/mL (*p* ≤ 0.05) (Figure 9B). We also used the supernatant from the cell culture to perform the PFU in the Vero 76 cell line. We observed a concentration-dependent inhibition of cytopathic effect formation in the Vero 76 cell culture. At a concentration as low as 0.0001 g/mL (EC_50_), a 50% decrease in PFU was observed, compared to HHV-1-infected controls (Figure 9C).

**Figure 10:**
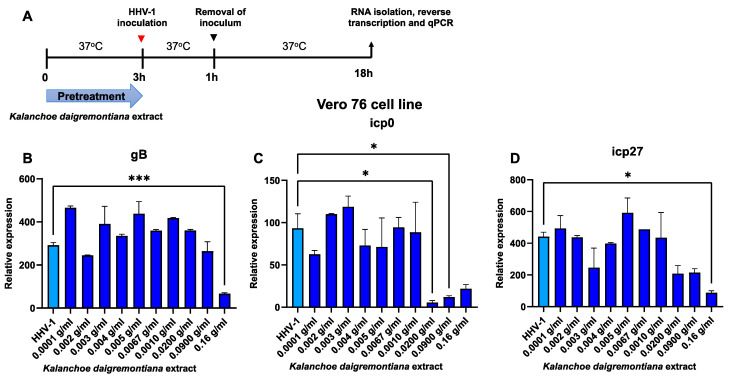
Extract from *K. daigremontiana* affects expression of immediate early and late HHV-1 genes in Vero 76 cells. Schematic representation of test procedures (**A**). Relative expression of *gB* (**B**). Relative expression of *icp0* (**C**). Relative expression of *icp27* (**D**). Data from three independent experiments are presented as mean ± SEM. Tukey’s multiple comparison test * *p* ≤ 0.05, and extremely significant at *** *p* ≤ 0.001.

**Figure 11:**
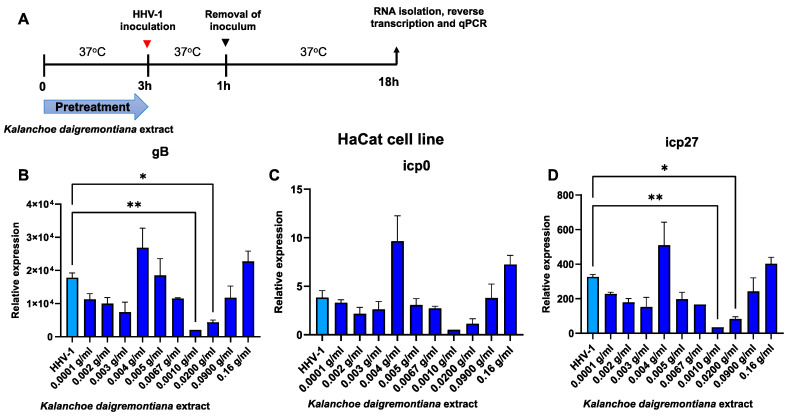
Extract from *K. daigremontiana* affects expression of immediate early and late HHV-1 genes in HaCat cells. Schematic representation of test procedures (**A**). Relative expression of *gB* (**B**). Relative expression of *icp0* (**C**). Relative expression of *icp27* (**D**). Data from three independent experiments are presented as mean ± SEM. Tukey’s multiple comparison test * *p* ≤ 0.05 or ** *p* ≤ 0.01.

As a next step, we decided to see if administration of the extract prior to infection would affect the relative expression of immediate early and late genes. Therefore, we examined the expression levels of three genes—*gB*, *icp0*, and *icp27* (Figure 10 and Figure 11).

For the Vero76 cell line, we observed a statistically significant decrease in the expression of the *gB* and *icp 27* genes for one of the extract concentrations used—0.16 g/mL (*p* ≤ 0.050), while, for the *icp0* gene, a statistically significant decrease in expression was observed at concentrations of 0.02 and 0.09 g/mL (*p* ≤ 0.050) (Figure 10).

On the other hand, in the HaCat cell line, a statistically significant decrease in *gB* and *icp27* gene expression was observed at concentrations of 0.01 g/mL (*p* ≤ 0.01) and 0.02 g/mL (*p* ≤ 0.05) (Figure 11).

**Figure 12:**
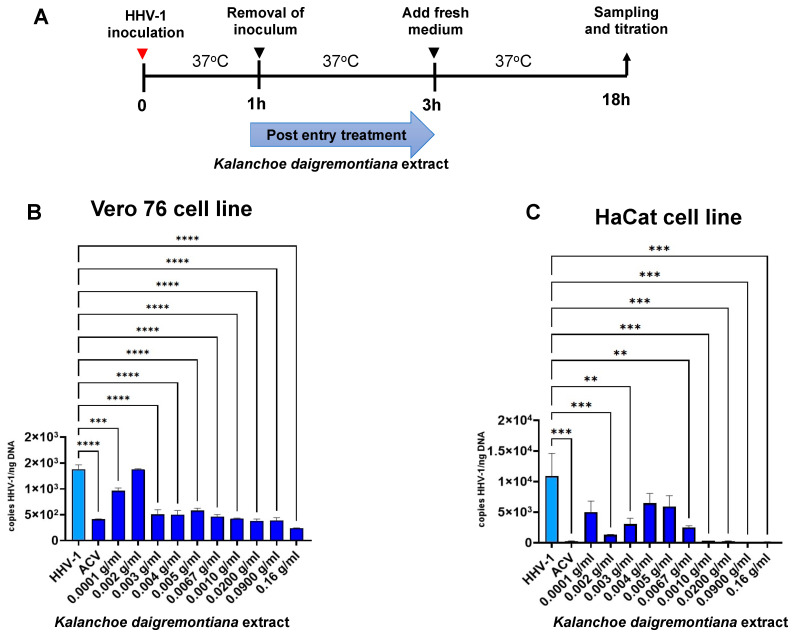
Efficiency of post-treatment with *K. daigremontiana* extract. Schematic representation of test procedures (**A**); Vero 76 cell line (**B**) and HaCaT cell line (**C**). At 24 h p.i., cells were subjected to HHV-1 copies titration by qPCR. Data from three independent experiments are presented as mean ± SEM. Tukey’s multiple comparison test ** *p* ≤ 0.01, and extremely significant at *** *p* ≤ 0.001 or **** *p* ≤ 0.0001.

In further experiments, we decided to investigate how the *K. daigremontiana* extract affects the inhibition of HHV-1 replication after virus entry into the cells. Both in Vero 76 and HaCaT cell lines, we observed a decrease in viral replication during post-entry treatment. We found a stronger decrease in the HaCaT cell line (Figure 12C) of more than 90% within the concentration range of 0.0067–0.16 g/mL (*p* ≤ 0.001). For the Vero cell line, we observed a dose-dependent decrease in replication with increasing extract concentrations, starting at 0.03 g/mL (*p* ≤ 0.0001) (Figure 12B).

Based on the obtained results, we calculated the effective concentration 50 (EC_50_) and selectivity index (SI) of the *K. daigremontiana* extract (Table 2). 

The concentrations of *K. daigremontiana* extract required to inhibit 50% of viral infection (EC_50_) and to induce 50% cell death (CC_50_) were determined in the 24 h assays. *K. daigremontiana* antiviral activities (EC_50_) for the tests ranged from 0.0028 μg/mL to 0.00184 μg/mL for the Vero76 cell line, while the value obtained for CC_50_ was 0.69 μg/mL. The EC_50_ values for the HaCat cell line ranged from 0.0067 to 0.0319, with a CC_50_ value of 0.48 g/mL (Table 2). 

The resulting selectivity index (SI) (the ratio of CC_50_ to EC_50_) for the *K. daigremontiana* extract ranged from 37.5 to 246.42 for the Vero 76 cell line and from 31.3 to 71.64 for the HaCat cell line (Table 2). 

**Figure 13:**
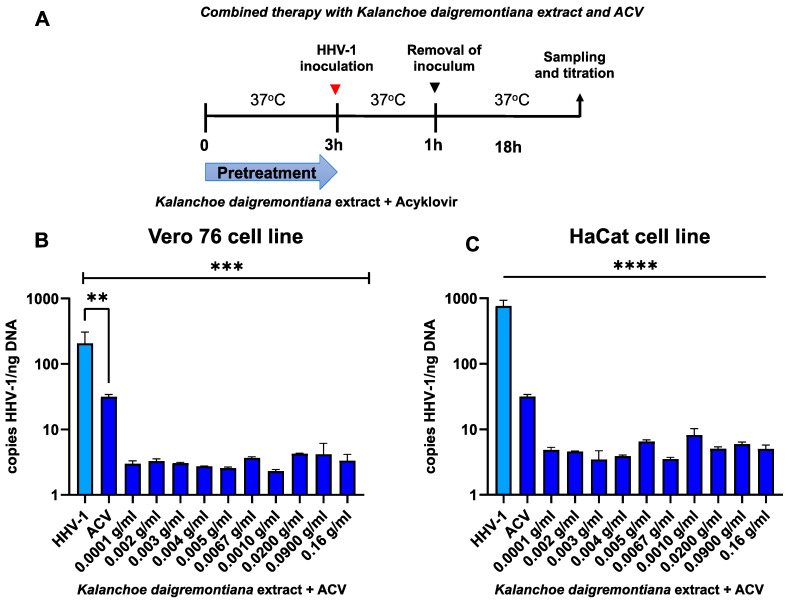
Efficiency of combined therapy (pretreatment) with *K. daigremontiana* extract and ACV. Schematic representation of test procedures (**A**); Vero 76 cell line (**B**) and HaCaT cell line (**C**). At 24 h p.i., cells were subjected to HHV-1 copies titration by qPCR. Data from three independent experiments are presented as mean ± SEM. Tukey’s multiple comparison test ** *p* ≤ 0.001, and extremely significant at *** *p* ≤ 0.001 or **** *p* ≤ 0.0001.

**Figure 14:**
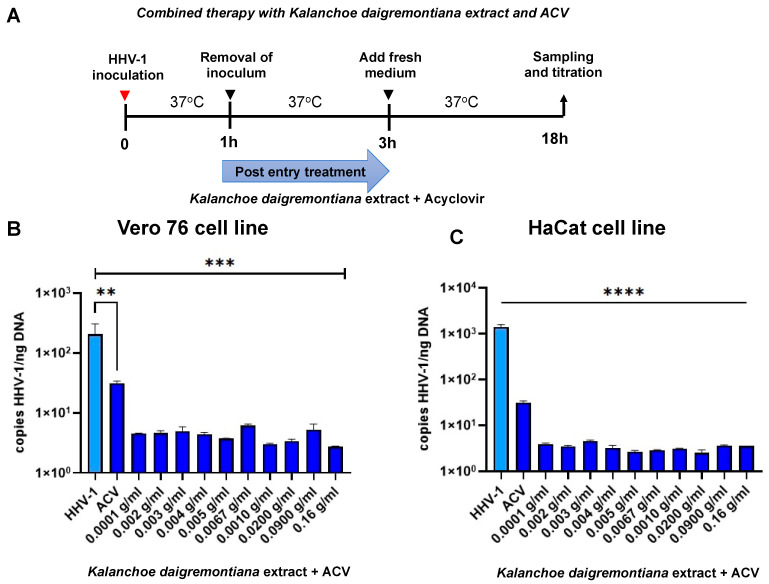
Efficiency of combined therapy (post-entry treatment) with *K. daigremontiana* extract and ACV. Schematic representation of test procedures (**A**); Vero 76 cell line (**B**) and HaCaT cell line (**C**). At 24 h p.i., cells were subjected to HHV-1 copies titration by qPCR. Data from three independent experiments are presented as mean ± SEM. Tukey’s multiple comparison test ** *p* ≤ 0.001, and extremely significant at *** *p* ≤ 0.001 or **** *p* ≤ 0.0001.

**Figure 15:**
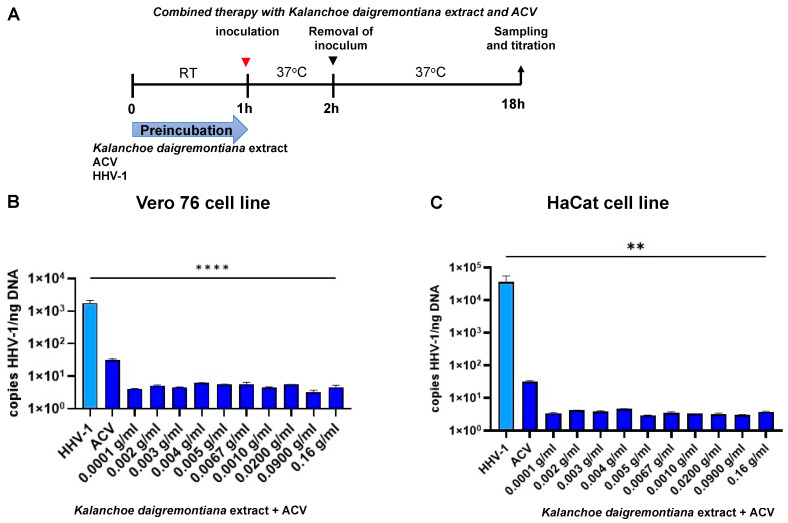
Efficiency of combined therapy (preincubation) with *K. daigremontiana* extract and ACV. Schematic representation of test procedures (**A**); Vero 76 cell line (**B**) and HaCaT cell line (**C**). At 24 h p.i., cells were subjected to HHV-1 copies titration by qPCR. Data from three independent experiments are presented as mean ± SEM. Tukey’s multiple comparison test ** *p* ≤ 0.001 and extremely significant at **** *p* ≤ 0.0001.

We also decided to test combination therapy (Figure 13, Figure 14 and Figure 15) with simultaneous treatment with acyclovir and *K. daigremontiana* extract. To check the effectiveness of the proposed therapy, we performed pretreatment, post-entry treatment, and preincubation tests.

The combination therapy with acyclovir was based on the administration of *K. daigremontiana* extract with ACV to cell lines 3 h before infection (pretreatment) (Figure 13). In the Vero 76 cell line (Figure 13A), we observed a statistically high decrease in viral replication for all concentrations of *K. daigremontiana* extract with acyclovir (*p* ≤ 0.001). In addition, administration of acyclovir alone proved less effective (*p* ≤ 0.001) compared to cells additionally treated with *K. daigremontiana* extract. In the HaCat cell line, we also noted a statistically high decrease in viral replication for all concentrations of the plant extract in combination therapy with acyclovir. 

We also decided to see what the efficacy of combination therapy was after viral infection. For this purpose, the cell lines were infected with HHV-1 and then treated 3 h later (post-entry treatment Figure 14). In both Vero76 and HaCat cell lines, we noted a highly statistically significant decrease in viral replication (*** *p* ≤ 0.001, **** *p* ≤ 0.0001) compared to cells untreated with combination therapy.

In addition, we decided to test the model for the preincubation of ACV with *K. daigremontiana* extract for one hour at room temperature. We wanted to note whether preincubation could influence better drug absorption by the cells (Figure 15). In both the Vero 76 and HaCat cell lines, we observed a significant decrease in HHV-1 replication, which was more statistically significant in the Vero76 cell line (*p* ≤ 0.0001) (Figure 15B).

### 2.4. The High-Content Imaging Screening Assay

**Figure 16:**
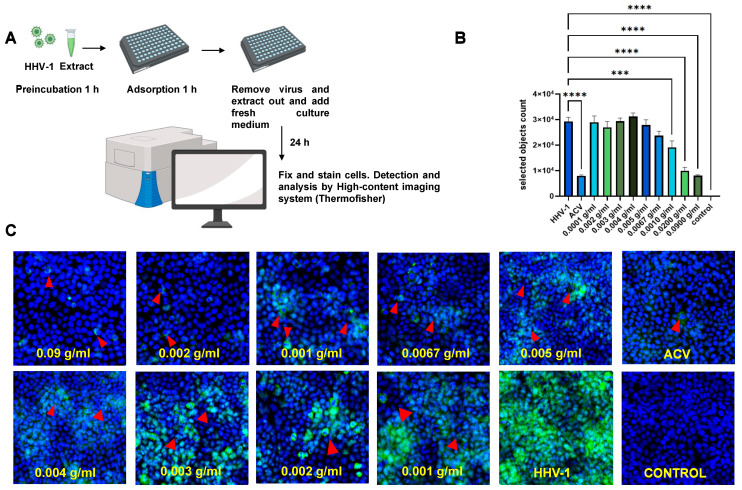
High-content imaging screening assay for HHV-1 antigens after treatment with the *K. daigremontiana* extract. Schematic representation of test procedures (**A**); dose-dependent anti-HHV-1 effects of *K. daigremontiana* extract using HCS spot detector protocols (the software counts the spot corresponding to the viral antigens) (**B**); representative images from analysis (green—HHV-1—red arrows; blue—DNA) (**C**), magnification 20×. Data from three independent experiments are presented as mean ± SEM. Tukey’s multiple comparison test at extremely significant at *** *p* ≤ 0.001 or **** *p* ≤ 0.0001.

The high-content imaging screening assay (Array Scan XTI, Thermofisher, Norristown, PA, USA) was developed and optimized in HaCaT cells infected with HHV-1 at 10^6^ PFU/mL. Acyclovir (4 µg/mL) was used as a reference drug. Different concentrations of *K. daigremontiana* extract were preincubated with HHV-1 for 1 h. The mixtures (virus and extract) were then applied for 1 h of adsorption (Figure 16A). Twenty-four hours post-infection, cells were fixed and stained with the primary antibody specific to major glycoproteins present in the viral envelope. A significant decrease in the amounts of viral antigens was observed in the range of extract concentrations from 0.001 to 0.09 (*p* ≤ 0.001). Incubation at a concentration of 0.09 induced the highest decrease in viral antigen presence (*p* ≤ 0.0001), as did the reference drug ACV. Higher concentrations of extract from *K. daigremontiana* also resulted in a decrease in viral antigen; however, they were not statistically significant. The HCS assay confirmed this in previous in vitro studies (Figure 16). 

## 3. Discussion

The genus *Kalanchoe* includes plants belonging to the *Crassulaceae* family, mainly succulents living in Madagascar, Africa, Asia, and South America. In Madagascar, they occur as endemics, and are characterized by different CAM-type photosyntheses: Crassulacean acid metabolism, which is an adaptation to arid ecosystems [17,18]. Natural substances contained in plants constitute an important group of compounds with antiviral properties, including anti-herpesviral activity. Many compounds such as phenols, glycosides, alkaloids, saponins, steroids, and tannins that affect HHV-1 replication have been described in the literature [9,10]. 

Extracts from plants belonging to the genus *Kalanchoe* are rich in numerous compounds including bufadienolides, bioactive flavonoids, and phenolic compounds. In addition, the presence of kaempferol 3-p-coumaroylarabinoside, named bryophylloside, is characteristic of *K. daigremontiana* extract [5,19,20,21]. The antiviral activity of kaempferol and its derivatives was shown for SARS-CoV-2 [22]. Interestingly, kaempferol inhibited SARS-CoV-2 invasion both in vitro and in vivo, mainly by binding to the SARS-CoV-2 S2 subunits and inhibiting viral fusion [22]. Furthermore, kaempferol-3-O-rhamnoside was shown to reduce both in vitro and in vivo infection; inhibition of HHV-1 induced brain injury was achieved by a reduction in microglial pro-inflammatory factors [23]. Apigenin isolated from *Ocimum basilicum* (OB), has shown similar virucidal activity for HHV-1 and HHV-2. Moreover, apigenin has also proven to be effective against ACV-resistant strains of HHV [24]. Quercetin is the most abundant dietary flavonoid, also present in *Kalanchoe* extract, and it has been shown to inhibit transcription and translation of viral proteins [6,9,10]. Lee et al. [25] demonstrated that the anti-HHV-1 effects of quercetin are related to the suppression of TLR-3-dependent inflammatory responses in Raw 264.7 monocytes through the inhibition of inflammatory transcriptional factors (NF-κB and IRF3) [26]. Gallic acid is the simplest phenolic acid, which may form esters with other flavonoids, but it also makes up the basis of hydrolysable tannins [6,7,8]. The antiviral activity of gallic acid and its esters was demonstrated to be related with virus attachment and penetration in classical plaque assays with HHV-1 [27]. The *K. daigremontiana* extract studied also contained the bufiandiols bryophyllin A and bersaldegenin-3-acetate, which showed an inhibitory effect on Epstein–Barr virus early antigen (EBV-EA) activation in Raji cells induced by the tumor promoter, 12-O-tetradecanoylphorbol-13-acetate. Bryophyllin A proved to be the most effective. The results also suggest the possibility of using *K. diagremontaiana* extract for other herpesviruses, belonging to a different family [28]. 

This study demonstrates anti-HHV-1 activity of water extract from *K. daigremontiana* in the in vitro infection of epithelial cells (Vero and HaCaT cell lines). *K. diagremontiana* extract inhibits different stages of HHV-1 infection, such as attachment and penetration, but also cell-to-cell infection (Figure 5, Figure 6, Figure 7, Figure 8, Figure 9, Figure 10 and Figure 11). Furthermore, we showed that the extract at selected concentrations reduces virus entry into the Vero 76 and HaCat cell lines. The antiviral activity of the polyphenols contained in plants depends on the chemical structure of the virus. The presence of phenolic groups, which exhibit antioxidant properties can have an inhibitory effect on the replication of viruses in host cells [29]. In addition, some polyphenols can block the structural domains of the receptors, making virus adsorption to the cell impossible. Our results demonstrated that *K. daigremontiana* water extract has the ability to prevent HHV-1 infection by direct inhibition of virus attachment, penetration, and blocking of infection when used pretreatment or post-treatment (Figure 5, Figure 6, Figure 7, Figure 8, Figure 9, Figure 10 and Figure 11). In the literature data, only one report is present in which the effect of compounds from *K. daigremontiana* extract against HHV-1 and HHV-2 were investigated. The authors of the study isolated individual fractions of the extract and tested the effects of the isolated flavonoids individually. The anti-herpesvirus activity was confirmed for two main flavonoids: kaempferol 3-O-β-d-xylopyranosyl-(1→2)-α-l-rhamnopyranoside and quercetin 3-O-β-d-xylopyranosyl-(1→2)-α-l-rhamnopyranoside [21]. However, these studies did not examine to what extent the components affect the replication cycle of the virus, whether they inhibit its entry (attachment, penetration, and pretreatment test) or the release of progeny virions (post-treatment). This appears to be crucial in assessing the usefulness of the tested compounds. The isolated compounds, or the entire extract, should be capable of mimicry, blocking host cell receptors or virus surface proteins to maintain entry into the cell. It is also useful to look for chemical groups in the structure of these compounds that may bind to the cell or virus surface to inhibit adsorption into the cell. Moreover, it seems to be more effective to use complete or fractionated extracts, so that we can have the synergistic and complementary effects of many antiviral compounds. It is worth noting that in the paper by Ürményi et al. [21], *K. daigremontiana* extract was subjected to flavonoid extraction, leading to isolation of two flavonoids, which showed an about ten times lower selectivity index compared to the extract. The SI in our study showed a similar value but differed depending on the tested cell line. Also, Sochocka et al. showed that *Ginko biloba* extract exhibits better anti-herpesvirus activity, compared to single polyphenols, such as quercetin and kaempferol [30]. 

Our data demonstrate that *K. diagremontiana* extract is a potent inhibitor of HHV-1 replication in keratinocytes and epithelial cells, which serve as the first line of defense during skin infections. However, the extract was much more effective for epithelial Vero cells than for HaCaT keratinocytes, and this effect correlates with the higher toxicity observed in HaCaT cells (the SI for the Vero 76 cell line reached higher values, compared to that of the HaCat cell line, Table 2). The toxicity of the extract may be based on the presence of volatile compounds, thus prompting us to employ further purification to obtain the extract of the flavonoids themselves.

Its inhibition of HHV-1 infection was similar to that of ACV, the most frequently chosen antiviral drug for humans. However, one must remember that the extract and ACV act at different stages of virus replication. ACV is an inhibitor of HHV-1 DNA replication, while for the extract, experiments showed inhibition of HHV-1 replication both during virus entry into the cells (pretreatment test) and during the release of progeny virions (post-treatment). It is worth mentioning that many studies indicate that *Kalanchoe* extract, in addition to its antiviral effects, exhibits anti-inflammatory and immunomodulatory effects, so we can influence the host’s antiviral response in a more complex manner [2]. More literature data are available on the anti-herpesviral properties of *Kalanchoe pinnata* extract. So far, the antiviral activity of the extract or its components against HHV-1, HHV-1, HCV, and EBV has been proven. Crayer et al. investigated the efficacy of two components contained in *Kalanchoe pinnata* extract against HHV-1, HHV-2, and Vaccinia virus. Both components, KB-100 and KB-200, showed activity against alphaherpesviruses and Vaccinia virus, but the KB-100 compound showed a stronger effect [4]. 

Our study also shows that the use of *K. daigremontiana* extract in combination with acyclovir significantly inhibits viral replication in both HaCat and Vero 76 cell cultures. Both pre-infection administration of the combination therapy and treatment of the infection were so effective that the level of viral replication did not exceed 10 copies/ng DNA.

Additional findings indicated that the *K. daigremontiana* extract acts as a post-entry inhibitor, likely affecting the stage between viral entry and replication of viral genomic DNA. During the HHV-1 life cycle, immediate early (IE), early (E), and late (L) genes are expressed once the viral DNA has entered the nucleus. The expression of IE genes is required for the transcription and subsequent expression of viral E and L genes, which play a critical role in the overall viral replication process. The study showed that *K. daigremontiana* extract reduced the expression of the *ICP0* and *ICP27* genes. These findings suggest that *K. daigremontiana* extract suppresses the expression of HHV IE genes by interfering with the expression of HHV-1 E and L genes, including glycoprotein B, which is critical for viral entry into cells. When the plant extract was administered prior to infection, it demonstrated a protective effect by reducing viral replication and inhibiting the expression of early and immediate early genes. A particularly strong therapeutic effect was observed in the HaCat cell line. Our hypothesis that the extract interferes with the processes between virus entry and exit from the cell is also supported by a study in which we used culture medium from over-infected cells treated with the extract in a pre-treatment assay to perform classical PFU in new cells. 

In addition, many studies indicate that *K. daigremontiana* extract or its individual components, such as quercetin, for example, influence the immune system. Studies have shown an enhanced antiparasitic immune response, using both in vitro and in vivo models. The anti-leishmanial, anti-inflammatory, anti-nociceptive, anti-edematogenic, wound-healing, and gastroprotective effects of flavonoids from *Kalanchoe* genus extract were demonstrated in mice and rats [31,32]. 

The presented results indicate the antiviral potential of the *K. diagremontiana* water extract, which opens future possibilities to test it in other viral models, as well as using in vivo model. 

## 4. Materials and Methods

### 4.1. Plant Materials and Extraction 

The extract was produced on an industrial scale in greenhouse conditions to protect plants from harmful environmental influences. The plants were not subjected to any chemical treatments that could have a detrimental effect on the extract and, thus, on human health. Direct exposure of leaves to the sun during cultivation was avoided in order to maintain their chlorophyll content. Sunlight was moderate and the temperature in the greenhouse did not fall below 15 °C, as *K. daigremontiana* is sensitive to low temperatures. The above-ground parts of *K. daigremontiana* were cut down and shredded, then placed in a tank extractor and subjected to a solvent extraction process with water. Undesired components of the original mixture were removed in a separate process. Name according to INCI: Aqua, *K. daigremontiana* Extract, Sodium Benzoate, Potassium Sorbate Ratio of herbal raw material to extract 4:1. The extract was preserved with a mixture of sodium benzoate (0.3%) and potassium sorbate (0.15%). 

### 4.2. UHPLC-DAD-MS Analysis

The analysis was conducted via UHPLC, utilizing the Ultimate 3000 series system (Dionex, Idstein, Germany), featuring a dual low-pressure gradient pump with a vacuum degasser, an autosampler, a column compartment, and a diode array detector coupled with an Amazon SL ion trap mass spectrometer (Bruker Daltonik GmbH, Bremen, Germany). Compound separation within the analyzed extracts was obtained on a Kinetex XB-C_18_ analytical column (150 mm × 2.1 mm × 1.9 μm) by Phenomenex (Torrance, CA, USA), with the column temperature maintained at 25 °C. Elution was performed using mobile phase A (0.1% formic acid in deionized water) and mobile phase B (0.1% formic acid in acetonitrile) via a multi-step gradient: starting at 1% B for 0 min, increasing to 30% B over 60 min, reaching 95% B at 90 min. The flow rate was set to 0.300 mL/min with 5 μL of the sample injected by the autosampler onto the column. Equilibration of the column was maintained for 10 min between injections. UV-vis spectra were recorded within the range of 200–450 nm, with chromatograms acquired at 280 and 350 nm. The eluate was directly introduced into the mass spectrometer without splitting. The ion trap Amazon SL mass spectrometer was equipped with an ESI interface, with the following parameters: nebulizer pressure of 38 psi, dry gas flow of 6.8 L/min, dry temperature of 134 °C, and capillary voltage of 4.5 kV. Analysis was conducted via scan from *m*/*z* 70–2200, with both compounds analyzed in negative ion mode. MS2 fragmentation was performed using Smart Frag mode. Detected compounds were identified based on UV-Vis and MS spectra in respect to existing literature reporting the chemical composition of *Kalanchoe* species and other closely related plants belonging to the *Crassulaceae* family. Elution order on reversed phase column was taken into account.

### 4.3. Cell Lines and Virus Strains

Human HaCaT keratinocytes were obtained from CLS Cell Lines Service GmbH (Eppelheim, Germany), while Vero 76 (CRL-1587) cells were purchased from ATCC (Washington, DC, USA). Both cell lines were cultured in Dulbecco’s modified MEM (DMEM) supplemented with 10% fetal calf serum, 10 U/mL of penicillin, and 100 μg/mL of streptomycin (Gibco by Thermo Fisher Scientific, Carlsbad, CA, USA). The cells were seeded into 24 well plates at a density of 1 × 10^4^/mL cells and cultured for 24 h before exposure to virus/extract, as described previously [12,13]. The McIntyre strain of HHV-1 was grown and titrated in Vero 76 cell cultures. The virus stocks (approximately 10^8^ PFU/mL) were stored at −80 °C. The multiplicity of infection (MOI) used in all experiments was 1. 

### 4.4. Cytotoxicity Assay

A cell proliferation reagent WST-1 (stable tetrazolium salt) assay was used to estimate the viability of the HaCaT and Vero 76 cell lines after treating the cells with *K. daigremontiana* water extract. The HaCaT and Vero 76 cells were seeded in 96-well plates at a density of 5 × 10^3^ cells/well and treated for 24 h with the *K. daigremontiana* water extract (dilutions of 0.0001–1 g/mL). After treatment, the cells were incubated with WST-1 (10 μL/well, Sigma Aldrich, Burlington, MA, USA) for 3 h. The absorbance (570 nm) of the solution was measured in the plate reader (Omega Microplate Spectrophotometer BioTek Instruments, Inc., Winooski, VT, USA). The results (±standard error mean (SEM)) were obtained from six repeats in three independent experiments. All the data were analyzed in GraphPad Prism version 9.5.0. 

### 4.5. Plaque Assay

Vero cells were maintained in a DMEM complete medium in 24-well plates until they reached full confluence (approximately 10^5^ cells per well). To evaluate the antiviral activity of *K. daigremontiana* extract, both the extract and HHV-1 (10^5^ PFU/mL) were incubated for 60 min at various extract concentrations. This mixture was then applied to the Vero cells and incubated for 60 min at 37 °C. Following this, the cultures were rinsed with cold PBS, and 2% methylcellulose in a complete culture medium was added to prevent viral spread through the culture medium. After 48 h, the plates were washed, stained with 1% crystal violet, and the plaques were counted. The same procedure was conducted with the cell culture medium from the cells infected and treated with *K. daigremontiana* extracts. The results were expressed as a percentage of plaque reduction inhibition, calculated using the following equation: 100 × [1 − (number of plaques with treatment/number of plaques without treatment)]. 

### 4.6. Antiviral Test 

HaCaT and Vero 76 cells were cultured in 24-well plates. To analyze how *K. daigremontiana* extract can influence viral attachment cells were pre-chilled at 4 °C for 15 min, then co-treated with *K. daigremontiana* extract and HHV-1 for 1 h at 4 °C. Next, inoculum was removed, and cell monolayers were washed with ice-cold PBS and further incubated at 37 °C. At 24 h post-infection, virus titers were determined by qPCR as described previously [12,13]. The viral penetration assay started by pre-chilling the cells at 4 °C for 15 min, and then the cells were infected for 1 h at 4 °C to allow virus binding but not entry. The inoculum was removed, and cells were washed with ice-cold PBS before adding *K. diagremontiana* extract for 2 h at 37 °C. The extract was afterwards removed, and cells were washed twice with cold PBS. After another 18 h at 37 °C, virus titers were determined by qPCR. To examine the post-entry treatment effects of *K. daigremontiana* extract use, cells were infected at 37 °C, then the virus was removed, cells washed, and extract were added 3 h post-infection (p.i.). At 24 h post-infection, the infected cultures were analyzed by qPCR. Pre-treatment was performed by incubating cells with *K. daigremontiana* extract (at 37 °C) for 3 h. Then, the cells were washed, infected, and further titered by qPCR. The positive control for all experiments was the reference drug acyclovir (ACV, 4 µg/mL). In the combination therapy, we treated the cell lines with both the plant extract and ACV (pretreatment, treatment, and preincubation acyclovir with *K. daigremontian* extract and HHV-1 were incubated for one hour and then added to the cell culture). The negative control consisted of uninfected cells. 

### 4.7. Real-Time PCR (qPCR)

For detection and quantification of HHV-1 DNA, a real-time PCR assay with a fluorescent TaqMan probe was used. DNA isolation was performed using a High Pure Viral Nucleic Acid Kit^®^ (Roche Diagnostics, San Jose, CA, USA), according to the manufacturer’s protocol. PCR was run on the LightCycler 480 instrument (Roche Diagnostics) with the modified in-house method described below [14]. A conservative region of HSV-1 genome encoding the viral glycoprotein B (gB) gene was chosen (GenBank: AB 297670), and a set of primers, as well as a probe labeled with the fluorophore reporter JOE on its 5′-end and with a BHQ-1 quencher on its 3′-end (Oligo^®^), were developed. Reaction was performed using TaqMan Master Kit^®^ (Roche Diagnostics). Final reaction mixture contained 5 µL of isolated viral DNA, 3.25 µmol/L of HSV1_A primer [5′-ATC CAC ACC TTA TCG TTT TTG T-3′], 3.25 µmol/L of HSV1_B primer [5′-CGT AAC GCA CGC TAG GGT-3′], and 1.50 µmol/L of HSV1_JOE probe [5′-JOE—GGC GGT TGG TCC AGA CGC–BHQ1-3′], in a total volume of 20 µL. Fluorescence levels were detected at 560 nm wavelength, specific for JOE fluorophore dye. 

RNA extracted from cells was transcribed into cDNA utilizing GoScript™ Reverse Transcriptase (Promega). qPCR assays for viral genes were performed using GoTaq^®^ Probe qPCR Master Mix (Promega) and specific primers as follows: gB_F: 5′TCTGCACCATGACCAAGTG3′; gB_R: 5′TGGTGAAGGTGGTGGATATG3′; ICP0_F: 5′GGTCCCCACTGACTCATACG3′; ICP0_R: 5′ATCCCGACCCCTCTTCTTC3′; ICP27_F: 5′TTCTCCAGTGCTACCTGAACC3′; ICP27_R: 5′TCAACTCGCAGACACGACTCG3′; GAPDH_F: 5′TGCACCACCAACTGCTTAGC3′;GAPDH_R: ′GGCATGGACTGTGGTCATGAG3′. 

The procedures were conducted according to the manufacturer’s guidelines using the QuantStudio™ 5 Real-Time PCR System (Thermo Fisher Scientific). The data were analyzed using the 2^−∆∆Ct^ method for quantifying relative gene expression.

### 4.8. High-Content Screening Assay (HCS)

The human keratinocytes cells were seeded in 96-well plates (at a density of 5 × 10^3^ cells/well), and treated with the water extract of *K. daigremontiana* at concentrations of 0.0001–1 mg/mL and infected with HHV-1. At 24 p.i., cells were fixed in 3.7% paraformaldehyde in PBS (Sigma-Aldrich) for 15 min in room temperature (RT). Next, the cells were permeabilized with 0.5% Triton X-100 (Sigma-Aldrich) in PBS (15 min in RT) and blocked with 1.5% bovine serum albumin (BSA, Sigma-Aldrich) in 0.1% Triton X-100 PBS solution (30 min) to prevent nonspecific binding. The presence of viral antigens was determined by direct IF, using FITC-conjugated Polyclonal Rabbit Anti-Herpes Simplex Virus 1 serum (Dako, dilution 1:200). Cell nuclei were stained with Hoechst 33258 (Sigma-Aldrich Chemicals Co., 2 µg/mL) in PBS. Non-infected cells were used as negative control and acyclovir-treated HHV-1-infected cells served as positive control. The fluorescent signals were detected and analyzed via high-content screening (Array Scan XTI, Thermo Fisher) at 10× magnification. The percentage of the infected cells in each well was automatically obtained from 9 images per well (8 wells per concentration) using HCS studio software version 2.0 using Spot detector protocols. 

### 4.9. Statistical Evaluation 

The results were statistically evaluated by one-way analysis of variation (ANOVA) followed by &Tukey’s multiple comparison test with GraphPad Prism™ version 9 software (GraphPad Software Inc., San Diego, CA, USA). Statistical differences were interpreted as significant at *p* ≤ 0.05 *, highly significant at *p* ≤ 0.01 **, and extremely significant at *p* ≤ 0.001 *** or *p* ≤ 0.0001 ****. 

## 5. Conclusions

In conclusion, we have demonstrated that *Kalanchoe daigremontiana* water extract shows the ability to prevent HHV-1 infection by direct inhibition of virus attachment, penetration, and blocking of infection when used in pretreatment or post-entry treatment. Our results indicate that *K. daigremontiana* extract may be a good candidate drug against HHV-1, both as a substance to prevent infection and to treat an already ongoing infection. Further studies should help to understand the role of particular extract compounds and their efficiency using an in vivo mouse model of infection. The authors believe that the extract should also be subjected to further purification to remove the toxic substances (such as some volatile compounds), as well as fractionation to detect the most effective fraction of flavonoids. Animal studies are also necessary to check how the *K. daigremontiana* water extract influences the immune response to HHV-1 infection. 

## Figures and Tables

**Table 1:** UHPLC-DAD-MS analysis of extracts obtained from *K. daigremontiana*.

N^o^	Compound Name	R_t_ [min]	UV-Vis Max [nm]	[M-H]^−^ *m*/*z*	MS² Ions (−)	Ref.
1	Undefined compound	3.3	268	267	221b	
2	Undefined phenolic acid derivative	11.2	300, 313	355	209, 191b	-
3	Undefined phenolic acid derivative	11.8	300sh, 312	355	209, 191	
4	Undefined compound	13.8	294	451	405b	-
5	Undefined phenolic acid derivative	15.6	225, 287sh, 312	355	337, 209, 191	-
6	Undefined compound	17.9	274	655	-	-
7	Undefined compound	20.9	230	431	385b, 205	-
8	Undefined compound	22.9	216, 292	449	287b, 269, 259	-
9	kaempferol 3-O-β-D-xylopyranosyl-(1→2)-α-L-rhamnopyranoside-7-O-β-D-glucopyranoside	27.9	264, 340	725	563b, 431, 413, 339, 285, 284, 255	[15]
10	Undefined compound	28.7	260	611	449, 431b, 251, 189	-
11	Undefined compound	31.5	261	449	269b, 207	-
12	quercetin 3-O-α-L-arabinopyranosyl-(1→2)-α-L-rhamnopyranoside		256, 263sh, 343	579	475,447, 429, 411, 383, 355, 300b, 271, 255, 229	[16]
13	Daigremontianin isomer	41.2	298	531a	485b, 455	-
14	Bryophyllin a	42.3	299	517a	471b, 387	[2]
15	Kapinnatoside (kaempferol 3-O-α-L-arabinopyranosyl-(1→2)-α-L-rhamnopyranoside)	42.3	264, 341	563	460, 431, 413, 327, 309, 285, 284b, 255	[16]
16	Bersaldegenin-1-acetate	46.1	299	519a	473, 459, 441, 413b, 395, 369	[2]
17	Bersaldegenin-2-acetate	47.2	298	519a	473, 414, 305, 343b	[2]
18	Daigremontianin	48.4	298	531a	485b, 407	[2]
19	Undefined compound	50.8	269	971	791, 748b, 702, 634, 568, 478, 408	-
20	Undefined compound	51.5	227	327	292, 229b, 211, 171	-
21	Undefined compound	55.4	228	329	293, 229b, 211	-
22	Undefined compound	57.0	285	483	437, 377b, 333, 281, 237, 185	-
23	Bersaldegenin-1,3,5-orthoacetate	59.7	299	501a	339b	[2]

Rt, retention time; MS, mass spectra; a—[M+HCOOH-H]-; b—basic peak; sh—shoulder in UV-Vis.

**Table 2:** Antiviral activity of *K. daigremontiana* extract against HHV-1 in Vero 76 and HaCat cells.

Cell Line	Antiviral Test	CC_50_ ^a^	EC_50_ ^b^	SI ^c^
Vero 76	attachment	0.69	0.0095	72.63
	penetration		0.0184	37.5
	pretreatment		0.0062	111.29
	post-entry treatment		0.0028	246.42
HaCat	attachment	0.48	0.0153	31.3
	penetration		0.0067	71.64
	pretreatment		0.0319	15.04
	post-entry treatment		0.0069	69.56

^a^ CC_50_ cytotoxic concentration 50, ^b^ EC_50_ effective concentration 50, ^c^ SI (Selectivity index) = CC_50_/EC_50_.

## Data Availability

Data are contained within the article.
